# Long-Lasting Complete Remission in a Patient With Metastatic Metaplastic Breast Cancer Treated With Immune Checkpoint Inhibitor and Chemotherapy: A Case Report and a Review of the Literature

**DOI:** 10.7759/cureus.53419

**Published:** 2024-02-01

**Authors:** Aydah Al-Awadhi, Safia Alnaqbi, Alia Albawardi

**Affiliations:** 1 Department of Medical Oncology, Tawam Hospital, Al Ain, ARE; 2 Department of Pathology, United Arab Emirates University, Al Ain, ARE

**Keywords:** triple-negative invasive breast carcinoma, treatment of the breast cancer, complete stable remission, cancer immunotherapy, metastatic metaplastic breast cancer

## Abstract

Metaplastic breast cancer (MpBC) is a rare form of breast cancer known for suboptimal response to chemotherapy, high recurrence rate, poor prognosis, and limited treatment options. Recent studies have reported that MpBC has high expression of programmed death ligand 1 and tumor-infiltrating lymphocytes, indicating the potential effectiveness of immunotherapy (IO) in MpBC. In addition, several reports have demonstrated the activity of IO in MpBC. In this case report, we present a case of recurrent MpBC that achieved durable, rapid, complete remission with atezolizumab (anti-PD-L1) and nab-paclitaxel with a continued response even after discontinued therapy.

## Introduction

Metaplastic breast cancer (MpBC) is a rare form of breast cancer (BC) that is considered a heterogeneous group of invasive carcinomas that have squamous and mesenchymal differentiation and account for less than 1% of all breast cancers [1]. MpBC usually exhibits a triple-negative molecular phenotype and has aggressive behavior and poor outcomes compared with the standard triple-negative invasive ductal carcinoma [2]. MpBC presents as stage IV in approximately 10% of the patients, and up to 50% of patients with early-stage disease will develop distant metastases [3]. Limited data is available regarding the best treatment modalities for MpBC, including chemotherapy (CT) [4]. MpBC is usually approached similarly to triple-negative invasive ductal carcinoma. However, it is known to have a suboptimal response to CT. For example, in a study examining response to neoadjuvant CT and first-line CT in MpBC, the response rates were reported to be 18.2% and 8.3%, respectively [5]. Also, another study showed that MpBC had a lower response to neoadjuvant CT when compared to triple-negative breast cancer (TNBC; 12.5 vs. 75%), and none of the MpBC patients achieved pathologic complete remission (pCR) to neoadjuvant CT [6]. It is possible that the molecular alterations in MpBC, such as those in epithelial-to-mesenchymal transition, could be responsible for the lower response to CT in MpBC when compared to TNBC [7]. Therefore, better strategies and clinical trials are needed to define effective approaches and targeted treatments for MpBC.

Immune checkpoint inhibitors (ICI), including anti-PD1 and anti-PD-L1, have emerged as an effective treatment strategy in combination with CT for early and metastatic TNBC [8]. Studies have shown that TNBC exhibits a greater presence of infiltrating lymphocytes (TILs), thereby establishing a favorable immune microenvironment for the potential use of ICIs [8-10]. In addition, it demonstrated a relatively higher tumor mutation burden (TMB), thus offering an antigenic foundation for immune cell recognition. Its elevated expression of PD-L1 (a protein expressed by tumor cells that can inhibit cytotoxic lymphocyte activity) presents a promising target for ICIs [7, 9, 10]. Currently, ICIs in combination with CT is the standard of care in the neoadjuvant setting for patients with stage II and above TNBC per the Keynote-522 clinical trial (NCT03036488) and in the first line setting in PD-L1 positive metastatic TNBC per the Keynote-355 clinical trial (NCT02819518) [11, 12]. However, these trials excluded patients with MpBC; therefore, the effectiveness of ICIs in MpBC is unknown. In a study that investigated comprehensive profiling of MpBC, increased PD-L1 expression was noted when compared with TNBC (46% vs. 9%, not otherwise specified, p<0.001) [13]. In addition, TILs are frequently observed in MpBC, and strongly PD-1-positive TILs have been described in half of the PD-L1-negative MpBC, proving its immunogenic phenotype, which may result in a potential increased effectiveness of ICIs in this entity of breast cancer [13, 14]. Here, we present a case of metastatic MpBC with a durable, complete response to ICIs.

## Case presentation

Demographic information and clinical findings

The patient was a 37-year-old premenopausal female of East Indian origin with no significant medical history. She initially felt a growing lump in her right breast during the second trimester of pregnancy in October 2019. She delivered a healthy baby girl in February 2020 and was referred to Tawam Hospital in May 2020. Physical examination noted a non-tender large right breast mass at the 5:00 position with overlying skin invasion and nipple inversion. No palpable right-sided axillary adenopathy was noted.

Diagnostic assessments

The mammogram showed a huge, circumscribed mass lesion measuring 66 x 103 x 123 mm occupying most of the right breast and associated with breast edema with small dense axillary lymph nodes seen in the axillary tail. Ultrasound of the right breast showed a complex cyst measuring 133 x 57 x 131 mm with internal mural growths, septations with moving internal echoes, and four small hypoechoic lymph nodes in the right axillary tail.

**Figure 1:**
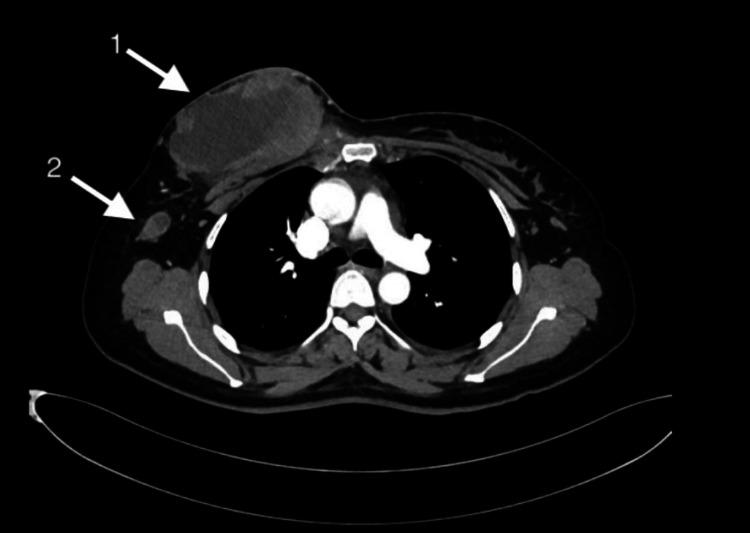
Computed tomography showing a large mass occupying all quadrants of the right breast (arrow 1) with large necrotic axillary lymphadenopathy (arrow 2)

Using computed tomography and bone scintigraphy, staging scans showed no distant metastatic disease. Computed tomography revealed a large, partially necrotic mass in the upper part of the right breast. The mass involves the full thickness of the medial aspect of the right pectoralis muscle. In addition, multiple large partially calcified and necrotic right axillary lymph nodes, the largest 21 X16 mm (Figure 1).

**Figure 2:**
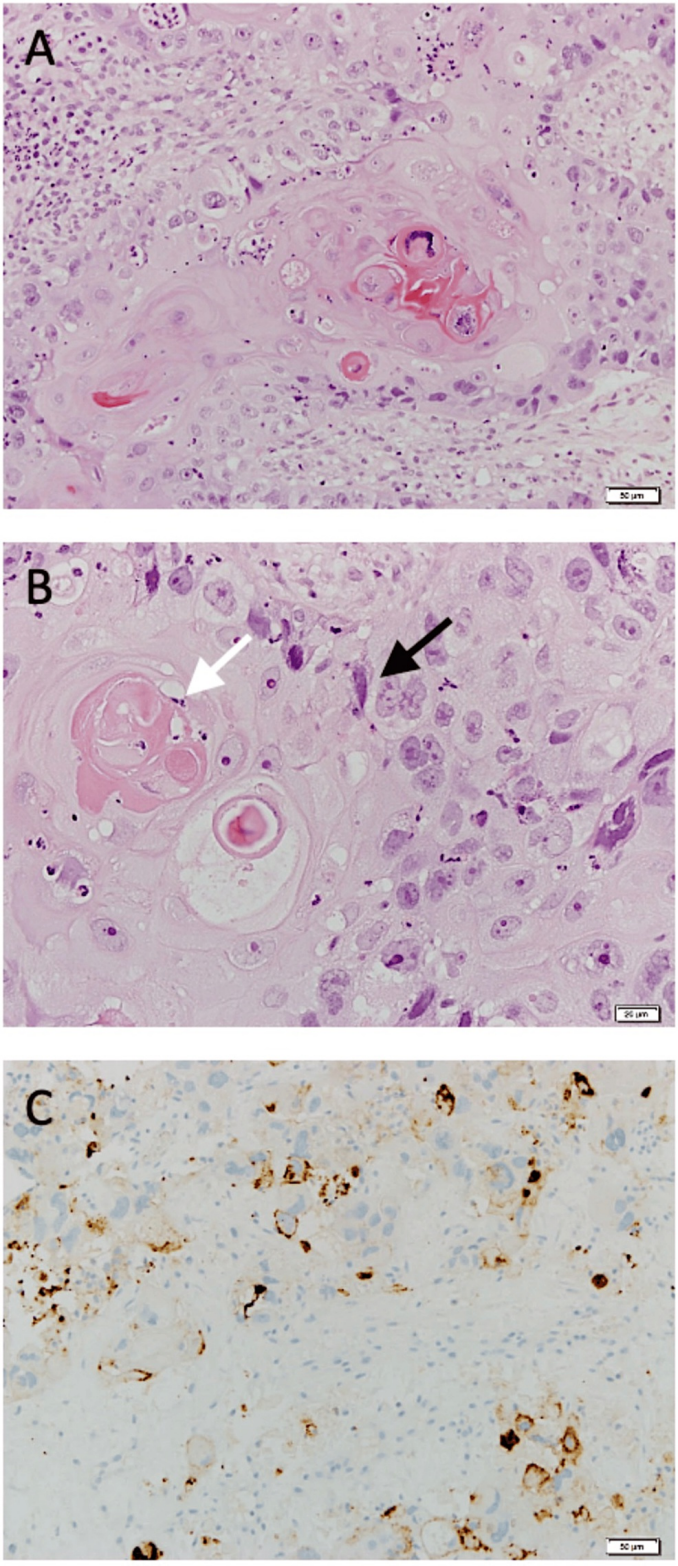
(A) Infiltrative tumor nests display squamous differentiation and marked pleomorphism (H&E stain, 20X); (B) Well-differentiated squamous cell carcinoma. Squamous differentiation is evident in the form of keratin pearls (white arrow) and intercellular bridges (black arrow) (H&E stain, 40X); (C) PDL1 immunostaining was performed (Ventana Ab SP142, FDA-approved kit). An adequate number of tumor cells is identified. The immune cells focally show PDL 1 expression - the score is 1.5% (20X)

Core biopsy of the right breast mass revealed histology consistent with grade 3 metaplastic carcinoma, and immunohistochemistry (IHC) showed estrogen (-), progesterone (-), HER-2 (0), P53 (+++), smooth muscle myosin heavy chain (+++), vimentin (+), and Ki-67 75% (Figure 2). Two core biopsies of the right axillary lymph node were negative for metastatic disease. The final clinical stage was cT4bN0M0.

Germline genetic testing revealed no pathogenic or likely pathogenic variants and neither variants of unknown significance (VUS) despite a strong family history of breast cancer in the mother and sister.

Therapeutic interventions and follow-up

The case was discussed in our breast multidisciplinary tumor board, and the decision was for upfront surgery. On June 1, 2020, the patient completed a right-modified radical mastectomy with axillary lymph node dissection. Intraoperatively, the tumor was noted to invade the pectoralis muscle. The patient recovered well postoperatively with no complications.

Postoperative pathology revealed a large fungating breast tumor measured 100 x 80 x 50 mm extending to the skin, nipple, and skeletal muscle. Histopathology was consistent with metaplastic carcinoma with a predominant squamous cell carcinoma component. Ductal carcinoma in situ was present in approximately 3% of the tumor and was of high grade and solid and comedo architecture. Lymphovascular invasion was observed. The margins were all negative except for the deep resection margin. Two out of 6 lymph nodes excised were positive for micrometastatic disease, and the size of the largest metastatic deposit was 18 mm with 1.5 mm extranodal extension. The final pathologic stage was pT4bN1M0. Repeat molecular markers were similar to the presurgical biopsy.

The plan was to start the patient on adjuvant CT with dose-dense Adriamycin and cyclophosphamide, followed by paclitaxel upon full wound healing [15]. However, less than four weeks after surgery, and when adjuvant chemotherapy had not started yet, the patient developed a growing fungating tender bleeding mass on the surgical wound concerning locoregional recurrence. Biopsy of the lesions was consistent with recurrent metaplastic carcinoma. PD-L1 IHC performed (using Ventana Ab SP142, FDA-approved kit; Roche Diagnostics Corporation, Indianapolis, Indiana) showed positive expression with a score of 1.5 %. Microsatellite stable disease was noted on IHC.

**Figure 3:**
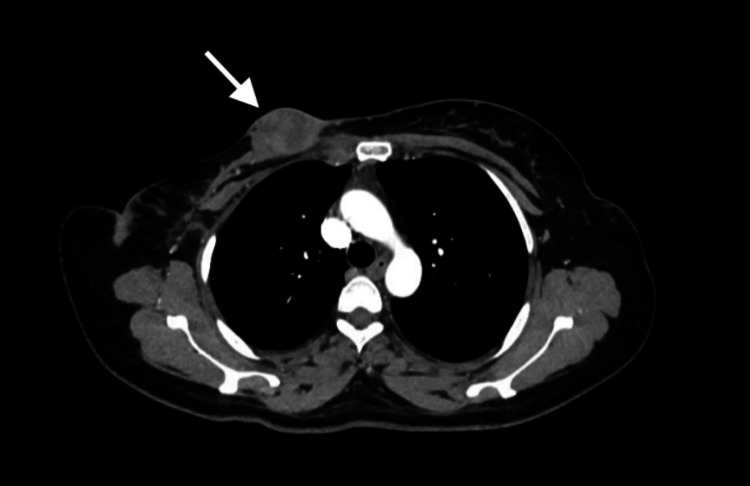
Horizontal section of computed tomography showing growing lesion on the surgical site invading the skin (arrow), indicating locoregional recurrence post right modified radical mastectomy

**Figure 4:**
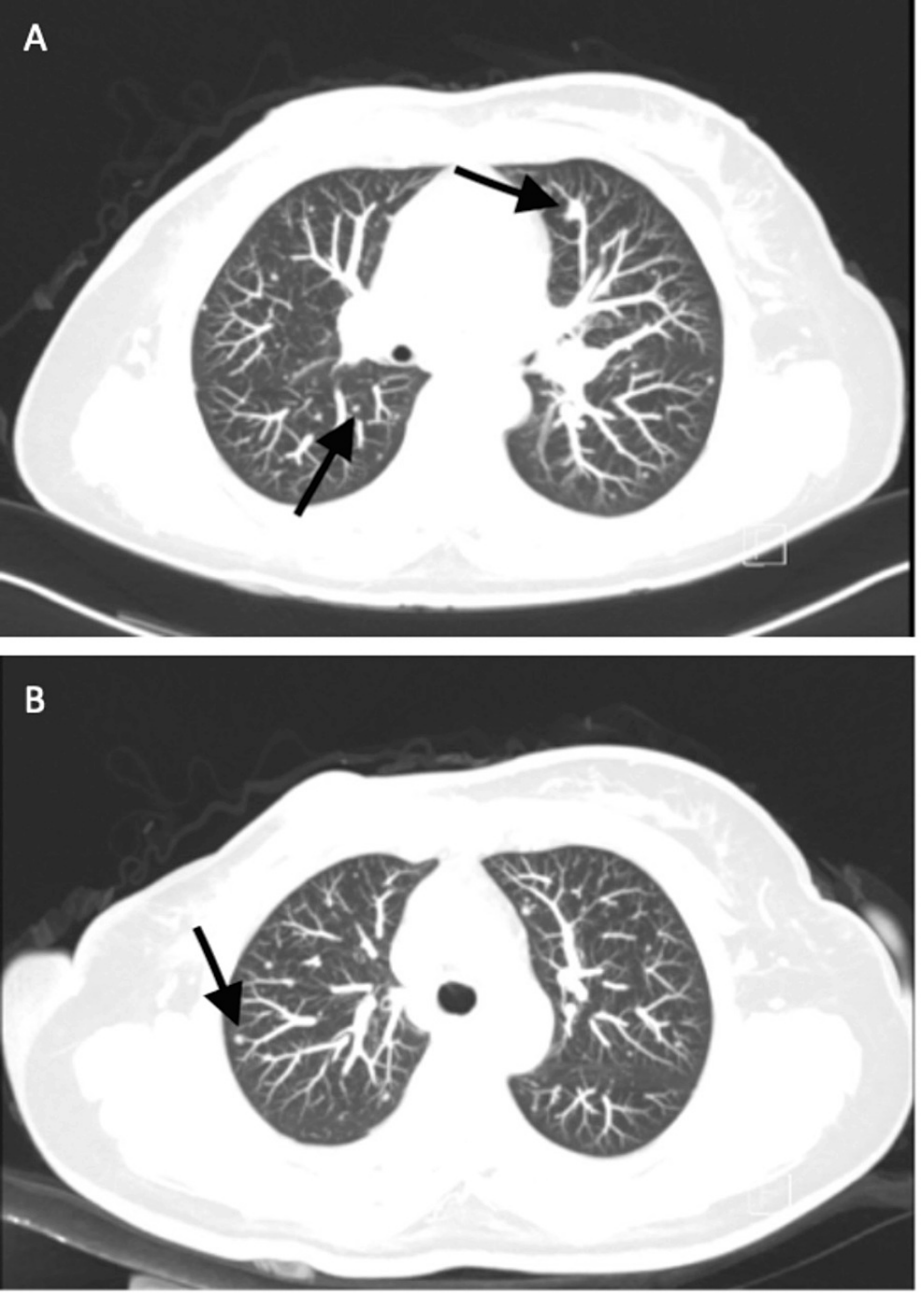
(A,B) Horizontal section of computed tomography showing widespread bilateral pulmonary nodules consistent with metastatic lung disease (arrows indicating some of the pulmonary nodules)

Staging scans with computed tomography done about six weeks from the initial pre-operative scans showed bilateral widespread pulmonary nodules consistent with metastatic lung disease in addition to a growing mass in the right chest wall (Figures 3-4).

After signing informed consent, the patient was administered atezolizumab (ICI that targets PD-L1) at a dose of 840 mg on days one and 15 intravenously and nab-paclitaxel at a dose of 100 mg/m^2^ of body surface area on days one, eight, 15 of every 28-day cycle intravenously. Treatment was started on July 8, 2020, with palliative intent. The treatment regimen was chosen per the Impassion-130 clinical trial as approved at that time by the FDA for first-line therapy of metastatic PD-L1 positive (score more or equal to 1% by Ventana Ab SP142) TNBC [16].

On examination at only the second week of therapy, the chest wall mass and bleeding have resolved completely with the appearance of granulation tissue. The patient was no longer with pain at the site. The patient was continued on the same therapy.

**Figure 5:**
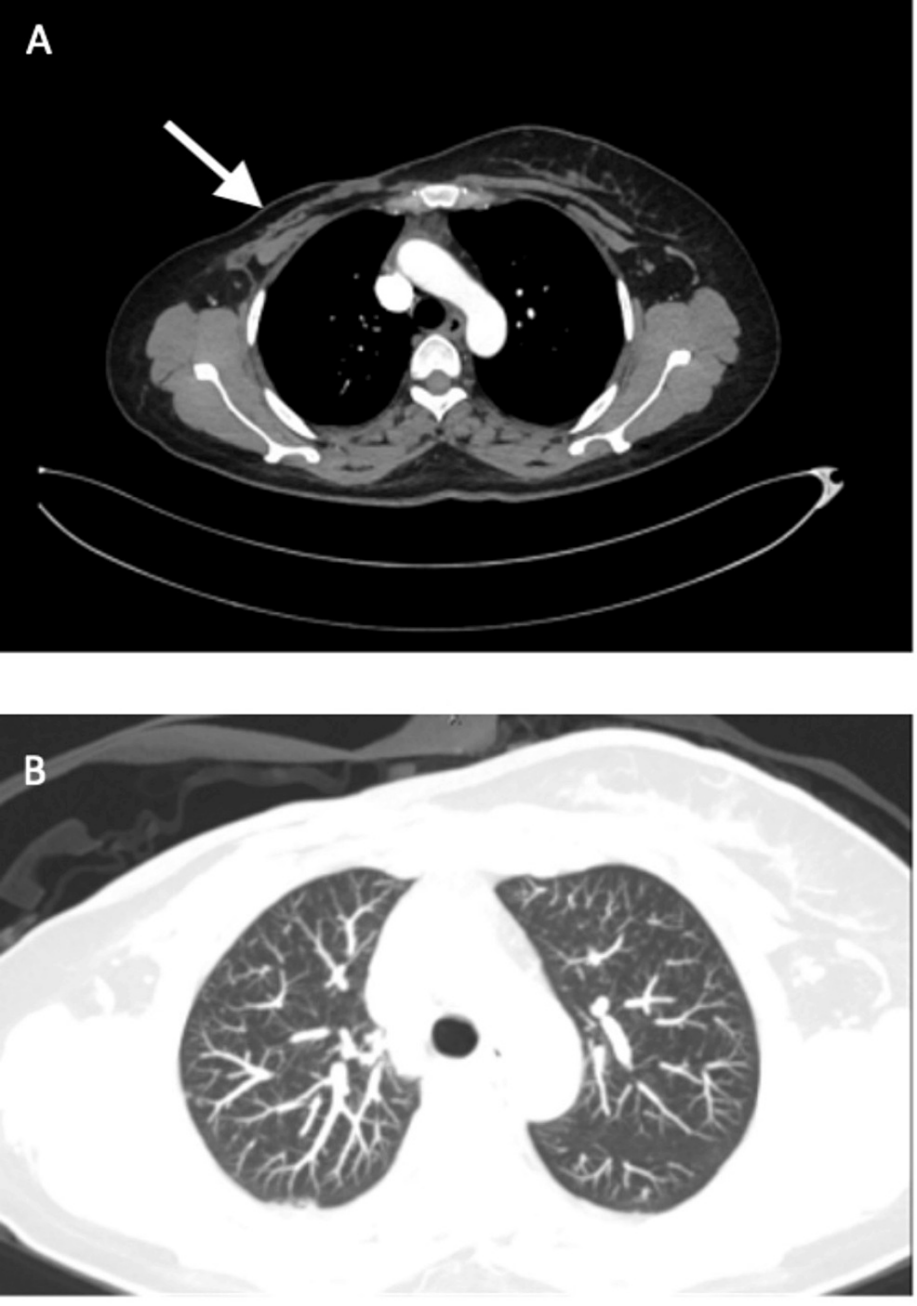
A horizontal section of computed tomography performed after three months of atezolizumab and nab-paclitaxel showed complete resolution of the right chest wall mass as indicated by the arrow (A) and complete resolution of bilateral pulmonary metastatic disease (B)

A repeat computed tomography scan three months after the start of therapy (on October 3, 2020) showed complete resolution of the soft tissue mass at the mastectomy bed and resolution of the numerous metastatic pulmonary nodules (Figure 5).

The patient tolerated treatment well, with no neuropathy or significant immune-related toxicity other than mild body itching without rash controlled with antihistamines.

On August 27, 2021, the manufacturer voluntarily withdrew the indication for atezolizumab and nab-paclitaxel for metastatic TNBC because of changes in the treatment landscape; despite this, however, the decision was made to continue the patient on the same therapy given the remarkable response [17].

After one year of therapy with atezolizumab and nab-paclitaxel, the patient remained in complete remission and had a good tolerance to therapy. To improve her quality of life and decrease frequent visits to the infusion center, we decided to drop nab-paclitaxel and continue atezolizumab at 1200 mg every three weeks as maintenance therapy.

After a total of two years of atezolizumab therapy, started in July 2020, she remained in complete remission with an excellent quality of life. The patient was keen to stop therapy and monitor, and indeed, after a lengthy discussion of the risks and benefits, therapy was stopped in August of 2022. The last computed tomography scan done when writing this article was in October of 2023 and showed no evidence of metastatic disease or interval change from previous scans. The patient continues to be followed up on regularly in our clinic. The patient continues to maintain a good quality of life with no long-term side effects from the therapy received.

## Discussion

According to the World Health Organization guidelines, MpBC is classified into various subtypes based on its cytopathological features and cell differentiation [1]. In this particular case, squamous cell differentiation may have contributed to the remarkable response to IO, as IO has been proven effective in squamous carcinomas across different tumor subtypes, such as cervical, lung, and head and neck cancers [18-21]. However, due to financial constraints, we lacked information on the expression of TILs and TMB in this case, which are important factors that could have shed light on the underlying mechanisms behind this dramatic response.

In our case, we observed a rapid and complete response to a combination of IO and CT as the initial treatment for metastatic MpBC.

The patient remained in remission even after one year of CT-IO combination on IO alone and even after stopping maintenance IO. Utilizing IO alone as a maintenance strategy post-CT and IO induction offers several advantages besides its safety profile and tolerability, which is more favorable than chemotherapy. For example, CT enhances tumor immunity by triggering immunogenic cell death (ICD), a regulated cellular demise that induces an inflammatory response leading to activation of cytotoxic T lymphocytes and an adaptive immune reaction [22]. Also, CT can disrupt the tumor's ability to evade the immune system by depleting suppressive immune cell populations [23].

It has been shown that different chemotherapeutic agents can elicit enhanced IO responses by different mechanisms [24]. Taxane-based chemotherapy was used in our case. Although its effect on stimulating the immune system is debatable, studies have shown that it can decrease the number and inhibitory function of the immunosuppressive cells, including regulatory T cells and myeloid-derived suppressor cells (cells that suppress the immune system), while maintaining the function of cytotoxic T cells [25-28]. For example, in one study, FoxP3 expression, a marker of T regulatory cells, was decreased in peripheral blood mononuclear cells when incubated with paclitaxel for a full day [28]. Moreover, a separate study demonstrated that following four cycles of neoadjuvant paclitaxel at a dosage of 200 mg/m2 administered every two weeks, along with subsequent surgical intervention, tumor analysis revealed immune infiltrates in approximately one-third of the patients [29]. This phenomenon was exclusively observed in patients who had exhibited a partial or complete response to the treatment, in contrast to the non-inflamed status of tumors prior to CT [29]. All these indicate that taxane was an appropriate choice to combine with IO.

Although the treatment was well-tolerated overall, the patient faced challenges with frequent visits to the infusion center. After two years of no evidence of disease, the patient expressed a desire to discontinue therapy. Our decision to stop therapy was supported by the growing evidence in the literature, indicating a low chance of disease relapse after discontinuing IO following a two-year treatment period, especially for those who have achieved and maintained complete remission [30, 31]. It is worth noting that re-challenging with IO is always an option in case of relapse. Most IO trials in the metastatic setting currently treat patients for two years, though other trials have continued IO treatment indefinitely [30-32].

Remarkably, in our case, there was no evidence of relapse after discontinuing CT and eventually stopping all treatments. 

Although MpBC is phenotypically triple-negative, it is biologically and genomically different from standard TNBC [5, 6]. Because of the rarity of MpBC, it has been treated as a variant of invasive ductal carcinoma. However, it has worse outcomes and overall survival, indicating the need for effective targeted options [4]. Several studies have confirmed the efficacy of IO combination therapies for TNBC [4]. More data is needed to elucidate the effectiveness of IO in MpBC [4].

Several studies have shown the process underlying the immunogenicity of MpBC. In an IHC study, tumoral PD-L1 expression, defined as ³5, was seen in 46% of the patients with MpBC when compared with other types, including 9% for patients with TNBC [13, 33]. Also, these tumors have high levels of TILs, which are indicative of an endogenous antitumor immune response [34]. The mechanism of these findings remains unclear; however, it provides a rationale for the utilization of IO in MpBC.

The first prospective trial of IO in MpBC (DART trial) showed that ipilimumab (CTLA-4 inhibitor) plus nivolumab (PD-L1 inhibitor) was clinically active in advanced MpBC with responses seen in 3/17 patients (ORR 18%). Importantly, all responses were durable (All responses are ongoing at >2 to almost three years later), which is unusual for MpBC and happened in highly CT refractory diseases. In the same study, responses were observed in PD-L1 negative or low expression, low TILs, and low TMB [35].

Encouragingly, several case reports have demonstrated a remarkably durable response to IO with and without CT in the metastatic setting and pCR in the early setting [36-40] (Table 1).

**Table 1:** List of case reports of remarkable responses to IO with and without chemotherapy in metaplastic breast cancer ICI - immune checkpoint inhibitors; TILs - infiltrating lymphocytes; TMB - tumor mutation burden; IO - immunotherapy

Case	Stage	Histology	ICI treatment	Line of treatment	Outcome	Duration of the response	PD-L1 expression in tumor cells	TILs	TMB
AlSayed et al. [36]	Recurrent stage IV	Metaplastic carcinoma with squamous cell	Durvalumab + paclitaxel (clinical trial protocol NCT02628132)	First-line metastatic	Complete response	>1 year and ongoing	20 percent	none	-
Gorchien et al. [37]	De-novo stage IV	Metaplastic carcinoma with epithelial and mesenchymal components along with area of bone formation	Pembrolizumab alone (clinical trial protocol NCT02447003)	First-line metastatic	Partial response	>2 years - ongoing	Positive	-	-
Fu et al. [38]	Recurrent stage IV	Metaplastic carcinoma with squamous cell carcinoma and sarcomatoid components	Toripalimab (PD-1 inhibitor) + anlotinib (multikinase tyrosine inhibitor)	Second-line metastatic	Partial response	>8 months - ongoing	CPS score <1% using the antibody 22C3	-	-
Ladwa et al. [39]	Stage II	Metaplastic carcinoma with malignant spindle cells embedded within a chondromyxoid stroma	Pembrolizumab + carboplatin + paclitaxel followed by Pembrolizuamb + Adriamycin + cyclophosphamide (Keynote-522 protocol)	Neoadjuvant	Pathologic complete remission	NA	TPS score 70%	-	-
Chen et al. [40]	Recurrent stage IV	Metaplastic carcinoma with a squamous component	Sintilimab + nab-paclitaxel	Second-line	Partial response	Progression after 20 months of therapy	-	-	12 muts/mb

A case series published in 2021 showed that three out of five patients with metastatic MpBC responded to ICIs, of which one had mixed metaplastic squamous carcinoma with invasive lobular carcinoma and low ER expression, which achieved complete response [41].

Overall, further studies are needed to identify an accurate predictive marker in MpBC to determine the response to IO.

## Conclusions

This case report describes a patient with advanced metastatic MpBC with a rapid, durable response in the form of complete remission after combined use of IO and chemotherapy. Although a prospective clinical trial is needed to confirm the benefit and safety of IO in MpBC, this case and other reported cases support the use of IO strategies in MpBC similarly to the standard TNBC. Further studies are also needed to identify a potential predictor of response to IO, including PD-L1 and TIL expression and TMB, to improve the survival outcome of such patients.

## References

[1] Tan PH, Ellis I, Allison K (2020). The 2019 World Health Organization classification of tumours of the breast. Histopathology.

[2] Basho RK, Gobbi H, Hennessy BT (2017). Metaplastic breast cancer. Textbook of Uncommon Cancer.

[3] Dave G, Cosmatos H, Do T, Lodin K, Varshney D (2006). Metaplastic carcinoma of the breast: a retrospective review. Int J Radiat Oncol Biol Phys.

[4] Shah DR, Tseng WH, Martinez SR (2012). Treatment options for metaplastic breast cancer. ISRN Oncol.

[5] Chen IC, Lin CH, Huang CS (2011). Lack of efficacy to systemic chemotherapy for treatment of metaplastic carcinoma of the breast in the modern era. Breast Cancer Res Treat.

[6] Jung SY, Kim HY, Nam BH (2010). Worse prognosis of metaplastic breast cancer patients than other patients with triple-negative breast cancer. Breast Cancer Res Treat.

[7] Reddy TP, Rosato RR, Li X, Moulder S, Piwnica-Worms H, Chang JC (2020). A comprehensive overview of metaplastic breast cancer: clinical features and molecular aberrations. Breast Cancer Res.

[8] Keenan TE, Tolaney SM (2020). Role of immunotherapy in triple-negative breast cancer. J Natl Compr Canc Netw.

[9] Heeke AL, Tan AR (2021). Checkpoint inhibitor therapy for metastatic triple-negative breast cancer. Cancer Metastasis Rev.

[10] Afkhami M, Schmolze D, Yost SE (2019). Mutation and immune profiling of metaplastic breast cancer: correlation with survival. PLoS One.

[11] Cortes J, Rugo HS, Cescon DW (2022). Pembrolizumab plus chemotherapy in advanced triple-negative breast cancer. N Engl J Med.

[12] Schmid P, Cortes J, Pusztai L (2020). Pembrolizumab for early triple-negative breast cancer. N Engl J Med.

[13] Joneja U, Vranic S, Swensen J (2017). Comprehensive profiling of metaplastic breast carcinomas reveals frequent overexpression of programmed death-ligand 1. J Clin Pathol.

[14] Ping Z, Siegal GP, Almeida JS, Schnitt SJ, Shen D (2014). Mining genome sequencing data to identify the genomic features linked to breast cancer histopathology. J Pathol Inform.

[15] Denduluri N, Somerfield MR, Chavez-MacGregor M (2021). Selection of optimal adjuvant chemotherapy and targeted therapy for early breast cancer: ASCO guideline update. J Clin Oncol.

[16] Schmid P, Adams S, Rugo HS (2018). Atezolizumab and nab-paclitaxel in advanced triple-negative breast cancer. N Engl J Med.

[17] (2021). Correction: Society for Immunotherapy of Cancer (SITC) clinical practice guideline on immunotherapy for the treatment of breast cancer. J Immunother Cancer.

[18] Li F, Zhai S, Lv Z (2022). Effect of histology on the efficacy of immune checkpoint inhibitors in advanced non-small cell lung cancer: A systematic review and meta-analysis. Front Oncol.

[19] Grau-Bejar JF, Garcia-Duran C, Garcia-Illescas D, Mirallas O, Oaknin A (2023). Advances in immunotherapy for cervical cancer. Ther Adv Med Oncol.

[20] Derman BA, Mileham KF, Bonomi PD, Batus M, Fidler MJ (2015). Treatment of advanced squamous cell carcinoma of the lung: a review. Transl Lung Cancer Res.

[21] Yu C, Li Q, Zhang Y, Wen ZF, Dong H, Mou Y (2022). Current status and perspective of tumor immunotherapy for head and neck squamous cell carcinoma. Front Cell Dev Biol.

[22] Galluzzi L, Vitale I, Warren S (2020). Consensus guidelines for the definition, detection and interpretation of immunogenic cell death. J Immunother Cancer.

[23] Heinhuis KM, Ros W, Kok M, Steeghs N, Beijnen JH, Schellens JH (2019). Enhancing antitumor response by combining immune checkpoint inhibitors with chemotherapy in solid tumors. Ann Oncol.

[24] Sordo-Bahamonde C, Lorenzo-Herrero S, Gonzalez-Rodriguez AP, Martínez-Pérez A, Rodrigo JP, García-Pedrero JM, Gonzalez S (2023). Chemo-immunotherapy: A new trend in cancer treatment. Cancers (Basel).

[25] Kodumudi KN, Woan K, Gilvary DL, Sahakian E, Wei S, Djeu JY (2010). A novel chemoimmunomodulating property of docetaxel: suppression of myeloid-derived suppressor cells in tumor bearers. Clin Cancer Res.

[26] Li JY, Duan XF, Wang LP (2014). Selective depletion of regulatory T cell subsets by docetaxel treatment in patients with nonsmall cell lung cancer. J Immunol Res.

[27] Roselli M, Cereda V, di Bari MG (2013). Effects of conventional therapeutic interventions on the number and function of regulatory T cells. Oncoimmunology.

[28] Zhang L, Dermawan K, Jin M (2008). Differential impairment of regulatory T cells rather than effector T cells by paclitaxel-based chemotherapy. Clin Immunol.

[29] Demaria S, Volm MD, Shapiro RL (2001). Development of tumor-infiltrating lymphocytes in breast cancer after neoadjuvant paclitaxel chemotherapy. Clin Cancer Res.

[30] Marron TU, Ryan AE, Reddy SM (2021). Considerations for treatment duration in responders to immune checkpoint inhibitors. J Immunother Cancer.

[31] Jansen YJ, Rozeman EA, Mason R (2019). Discontinuation of anti-PD-1 antibody therapy in the absence of disease progression or treatment limiting toxicity: clinical outcomes in advanced melanoma. Ann Oncol.

[32] Iivanainen S, Koivunen JP (2019). Early PD-1 therapy discontinuation in responding metastatic cancer patients. Oncology.

[33] Grabenstetter A, Jungbluth AA, Frosina D (2021). PD-L1 expression in metaplastic breast carcinoma using the PD-L1 SP142 assay and Concordance among PD-L1 immunohistochemical assays. Am J Surg Pathol.

[34] Tray N, Taff J, Singh B (2019). Metaplastic breast cancers: genomic profiling, mutational burden and tumor-infiltrating lymphocytes. Breast.

[35] Adams S, Othus M, Patel SP (2022). A multicenter Phase II trial of ipilimumab and nivolumab in unresectable or metastatic metaplastic breast cancer: cohort 36 of dual anti-CTLA-4 and anti-PD-1 blockade in rare tumors (DART, SWOG S1609). Clin Cancer Res.

[36] Al Sayed AD, Elshenawy MA, Tulbah A, Al-Tweigeri T, Ghebeh H (2019). Complete response of chemo-refractory metastatic metaplastic breast cancer to paclitaxel-immunotherapy combination. Am J Case Rep.

[37] Gorshein E, Matsuda K, Riedlinger G (2021). Durable response to PD1 inhibitor pembrolizumab in a metastatic, metaplastic breast cancer. Case Rep Oncol.

[38] Fu Y, Liu J, Jiang Y (2022). Partial response after Toripalimab plus Anlotinib for Advanced metaplastic breast carcinoma: a case report. Front Endocrinol (Lausanne).

[39] Ladwa A, Elghawy O, Schroen A, Abernathy K, Schlefman J, Dillon P (2023). Complete response of triple-negative metaplastic carcinoma of the breast using pembrolizumab. Case Rep Oncol.

[40] Chen L, Meng Z, Zhou Z (2023). Immunotherapy combined with chemotherapy in relapse metaplastic breast cancer. Onco Targets Ther.

[41] Kim I, Rajamanickam V, Bernard B (2021). A case series of metastatic metaplastic breast carcinoma treated with anti-PD-1 therapy. Front Oncol.

